# Microbial Diversity and Interaction Specificity in Kombucha Tea Fermentations

**DOI:** 10.1128/msystems.00157-22

**Published:** 2022-06-07

**Authors:** Elizabeth A. Landis, Emily Fogarty, John C. Edwards, Otilia Popa, A. Murat Eren, Benjamin E. Wolfe

**Affiliations:** a Department of Biology, Tufts University, Medford, Massachusetts, USA; b Committee on Microbiology, University of Chicago, Chicago, Illinois, USA; c Process NMR Associates, LLC, Poughkeepsie, New York, USA; d Department of Medicine, University of Chicago, Chicago, Illinois, USA; University of California—Berkeley

**Keywords:** *Brettanomyces*, *Komagataeibacter*, acetic acid bacteria, fermentation, kombucha, microbiome, yeast

## Abstract

Despite the popularity of kombucha tea, the distribution of different microbes across kombucha ferments and how those microbes interact within communities are not well characterized. Using metagenomics, comparative genomics, synthetic community experiments, and metabolomics, we determined the taxonomic, ecological, and functional diversity of 23 distinct kombuchas from across the United States. Shotgun metagenomic sequencing demonstrated that the bacterium Komagataeibacter rhaeticus and the yeast Brettanomyces bruxellensis were the most common microbes in the sampled kombucha communities. To determine the specificity of bacterium-yeast interactions, we experimentally quantified microbial interactions within kombucha biofilms by measuring densities of interacting species and biofilm production. In pairwise combinations of bacteria and yeast, *B. bruxellensis* and individual strains of *Komagataeibacter* spp. were sufficient to form kombucha fermentations with robust biofilms, but Zygosaccharomyces bisporus, another yeast found in kombucha, did not stimulate bacteria to produce biofilms. Profiling the spent media of both yeast species using nuclear magnetic resonance spectroscopy suggested that the enhanced ability of *B. bruxellensis* to ferment and produce key metabolites in sucrose-sweetened tea may explain why it stimulates biofilm formation. Comparative genomics demonstrated that *Komagataeibacter* spp. with >99% genomic similarity can still have dramatic differences in biofilm production, with strong producers yielding five times more biofilm than the weakest producers.

**IMPORTANCE** Through an integration of metagenomic and experimental approaches, our work reveals the diversity and nature of interactions among key taxa in kombucha microbiomes through the construction of synthetic microbial pairs. Manipulation of these microbes in kombucha has the potential to shape both the fermentation qualities of kombucha and the production of biofilms and is valuable for kombucha beverage producers, biofilm engineers, and synthetic ecologists.

## INTRODUCTION

**FIG 1:**
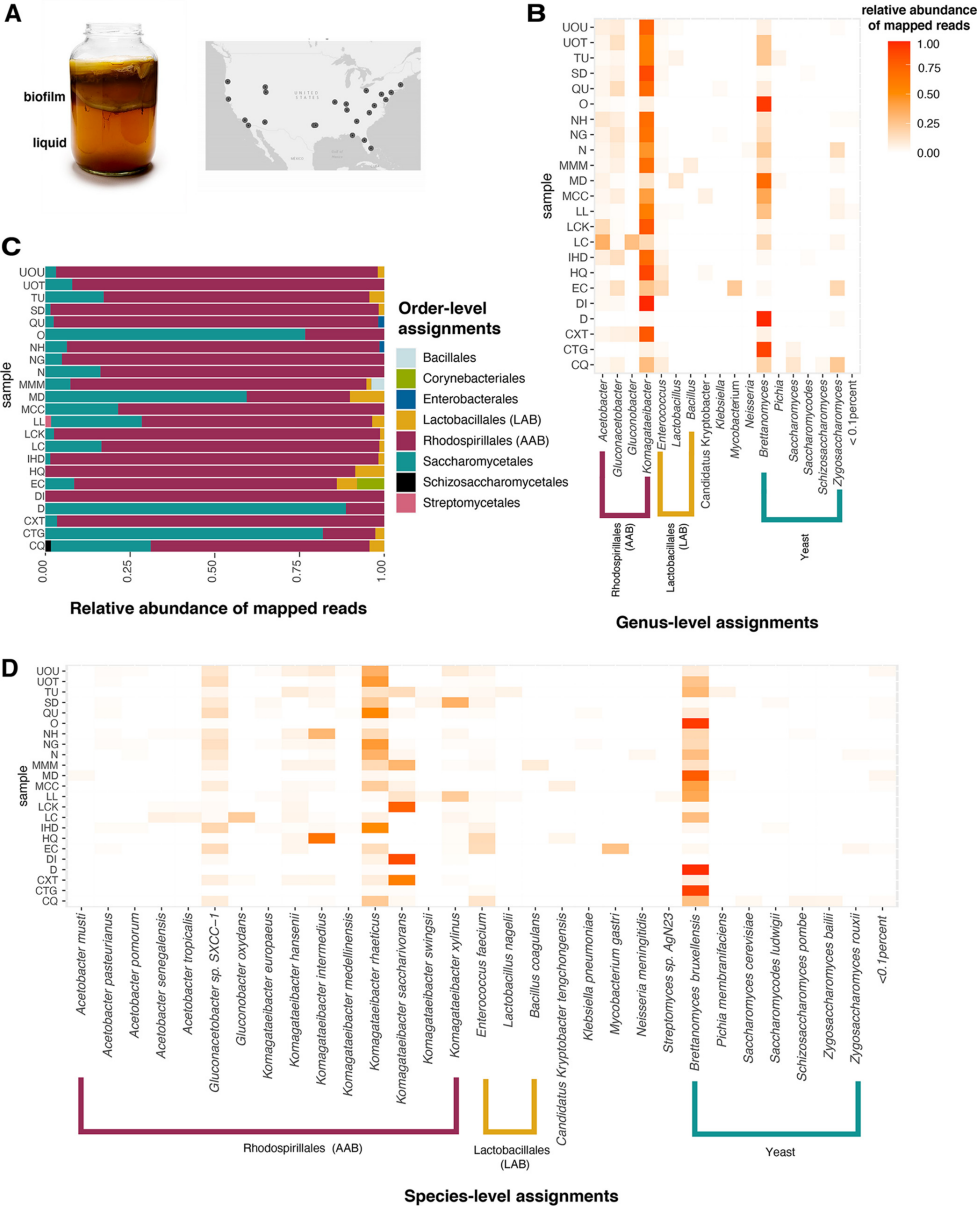
Taxonomic diversity of 23 kombucha microbiomes. (A) Kombucha fermentation, showing the floating biofilm and liquid tea (50). Samples of kombucha starter from across the United States, containing both biofilm and liquid, were acquired through the website Etsy.com or through donations, and the liquid portions were sequenced using shotgun metagenomic sequencing. Each black dot on the map represents the geographic origin of kombuchas sampled in this study. (B) Order-level taxonomies were assigned to unassembled reads using Kaiju and the NCBI BLAST nr+euk database, which contains bacteria, yeasts, viruses, and microbial eukaryotes. The orders *Saccharomycetales* (yeast), *Rhodospirillales* (AAB), and *Lactobacillales* (LAB) were the most frequent and abundant of classified reads. (C) Genus-level taxonomy assignments were obtained in the same manner. The genus *Komagataeibacter* was the most abundant AAB, *Enterococcus* the most abundant LAB, and *Brettanomyces* the most abundant yeast detected at the genus level. (D) At the species level, Komagataeibacter rhaeticus was the most common and abundant bacterium detected. *Brettanomyces bruxellensis* was the most common yeast detected. See Tables S1 to S3 for taxon tables.

Kombucha is a fermentation of tea and sugar that can be purchased in stores or made at home after obtaining a starter culture from a community or commercial source. The starter inoculum and the tea itself both contain acetic acid bacteria (AAB), yeast, and sometimes lactic acid bacteria (LAB) (1). Certain AAB present in kombucha produce bacterial cellulose, which forms a floating biofilm at the air-liquid interface during fermentation (Fig. 1A). This cellulosic biofilm is often included as part of the starter and is transferred along with some liquid to inoculate fresh batches of tea. Cellulose produced by AAB in kombucha is also of interest as a material for various applications, including artificial corneas, wound dressings, and biodegradable packaging (2–4). Despite growing popularity and perceived health benefits of kombucha (5), a comprehensive understanding of how different microbial species are distributed across unique kombucha fermentations (taxonomic diversity) and how these species interact within kombucha (ecological and functional diversity) is lacking. This knowledge gap limits our ability to predictably manage or engineer this culturally and economically significant microbial community.

10.1128/msystems.00157-22.5 TABLE S2 Relative abundance of other bacteria (including lactic acid bacteria [LAB]) at the species level across each kombucha ferment. Download Table S2, DOCX file, 0.02 MB. Copyright © 2022 Landis et al. 2022 Landis et al. https://creativecommons.org/licenses/by/4.0/ This content is distributed under the terms of the Creative Commons Attribution 4.0 International license.

A variety of studies have begun to capture the taxonomic diversity of kombucha biofilms, but due to differences in sampling and analysis approaches across studies, a clear picture of the taxonomic diversity of kombucha is still emerging (6–9). Previous studies have employed culture-based (10, 11) and metagenomic (6–9) analyses to quantify microbial diversity and have sampled different spatial components of kombucha tea. Across these studies, the AAB *Komagataeibacter* and *Gluconobacter*, and the LAB *Lactobacillus* are commonly identified bacterial genera and commonly identified fungal genera include the yeast *Brettanomyces* and Zygosaccharomyces. Given the compartmentalization between tea and biofilm within kombucha fermentations and wide variation in fermentation practices, additional sequencing of unique kombucha fermentations is needed to help confirm whether these taxa represent the core constituency of kombucha microbiomes.

While there is some previous work on patterns of kombucha taxonomic diversity, the ecological interactions and functional diversity of kombucha microbes are poorly characterized. The nature of interactions between AAB and yeast in kombucha is a source of frequent speculation and conjecture. Colloquially, the bacterial cellulose starter which is transferred (along with some liquid) during fermentation is known as a “SCOBY”: Symbiotic Community of Bacteria and Yeast. The “symbiotic” nature of this relationship is often assumed to be a mutualism, at least in part due to their complementary metabolism of sugars. AAB are not thought to efficiently utilize the sucrose supplied at the onset of fermentation. Instead, they metabolize breakdown products from yeast invertases, including glucose, fructose, and ethanol (12). Because of these purported interactions between bacteria and yeast, kombucha has been proposed as a model system to study microbial cooperation and conflict (13). However, few studies have directly examined interactions between yeast and bacteria in kombucha. Marsh et al. (8) posited that yeasts play a somewhat nonspecific and interchangeable role in kombucha fermentation. However, previous studies attempting to pair common yeasts and bacterial isolates from kombucha have failed to produce biofilms which are a hallmark of kombucha fermentation (12, 14). This suggests either that pairwise inoculations are not capable of producing kombucha-like fermentations or that only certain pairs of bacteria and yeast produce biofilms.

To obtain a deeper understanding of the microbial dynamics in kombucha fermentations, we first determined the taxonomic diversity of a set of 23 kombucha microbiomes from across the United States using shotgun metagenomic sequencing. We found that the yeast Brettanomyces bruxellensis and the bacterium Komagataeibacter rhaeticus were frequently detected in these fermentations. We then used this kombucha collection to isolate strains of dominant taxa and used these to measure outcomes of different AAB-yeast pairs. Only certain pairwise combinations of bacteria and yeasts were able to form robust biofilms and reach pH levels in the range of typical kombucha fermentations. Zygosaccharomyces bisporus, a yeast commonly reported in kombucha fermentations, failed to stimulate bacterial growth and biofilm production by *Komagataeibacter* isolates, while *B. bruxellensis* stimulated both. Growth measurements and metabolomics of yeast spent media suggest that *Z. bisporus* grows more slowly and produces fewer fermentation products than does *B. bruxellensis.* Different bacterial strains of *Komagataeibacter* used in coculture also led to differences in biofilm production, suggesting that strain-level variation among *Komagataeibacter* is an important variable shaping kombucha fermentations. Our work supports an emerging view of the core constituency of kombucha microbiomes, provides a framework for building synthetic kombucha biofilms, and suggests possible metabolic differences that may be driving variable outcomes of yeast-bacterium interactions in this fermentation system.

## RESULTS AND DISCUSSION

### Metagenomic sequencing identifies core species in kombucha ferments.

We first examined the microbial diversity of kombucha samples by assigning metagenomic short reads with taxonomic identities using Kaiju. At the order level (Fig. 1B), AAB (order *Rhodospirillales*) were present in every sample and were the most abundant read designations, making up on average 75% of classified reads.

The order *Lactobacillales* was detected in 13 of 23 communities and constituted 2.31% of samples on average (Fig. 1B). Yeasts in the order *Saccharomycetales* constituted 20.7% of reads classified at this level. (Fig. 1B) At the genus level, the yeast genera Brettanomyces and Zygosaccharomyces made up 25 and 3.4% of reads present, respectively (Fig. 1C). Overall, 13% of reads were unmapped and 2.1% could not be assigned at the order level.

On average, starters contained 5.9 species of AAB, 0.87 species of LAB, and 1.5 species of yeast. The most frequent and abundant bacterial species detected was K. rhaeticus, present in 23 of 25 samples at >1% abundance and composing on average 18% of classified reads (Fig. 1D; see also Tables S1 to S3 in the supplemental material). However, this may be an underestimate of the abundance of *K. rhaeticus* reads; *Gluconacetobacter* SP-SXCC1, a reference isolate from Chinese vinegar (on average 6.8% of reads classified) is correlated with the presence of *K. rhaeticus* (Pearson’s rho = 0.89, false discovery rate [FDR]-corrected *P* < 0.001) suggesting indiscriminate classification of reads between these two isolates. Indeed, *Gluconacetobacter* sp. strain SP-SXCC1 is likely a strain of *K. rhaeticus* based on 16S rRNA gene sequence similarity (15). Enterococcus faecium was the most frequent LAB detected (16 of 23 starters). The yeast *B. bruxellensis* was ubiquitous in our sampling and was the most frequently detected yeast (21 of 23 starters), with no other yeast species detected at >1% abundance in more than two starters. In two samples (HQ and DI), no yeast reads were detected, potentially due to a very low abundance of yeasts in these samples or due to a lack of a reference sequence for the yeast in these samples in the databases used in our analysis.

10.1128/msystems.00157-22.4 TABLE S1 Relative abundance of acetic acid bacteria (AAB) at the species level across each kombucha ferment. Download Table S1, DOCX file, 0.02 MB. Copyright © 2022 Landis et al. 2022 Landis et al. https://creativecommons.org/licenses/by/4.0/ This content is distributed under the terms of the Creative Commons Attribution 4.0 International license.

Our findings support the recent amplicon metagenomic study of kombucha by Harrison and Curtin (9), who found that the genera *Brettanomyces* and *Komagataeibacter* dominate SCOBYs; in that study, these genera were detected in 97 and 99% (respectively) of 103 commercial kombucha samples at >0.1% abundance. This congruence of findings is striking because our study used different approaches for sampling (liquid from fermentations in our study versus biofilm), sequencing (shotgun metagenomic sequencing in our study versus mostly amplicon sequencing), and analysis (Kaiju analysis pipeline in our study versus QIIME and Kraken). Combined, these two studies represent the most comprehensive sampling of kombucha taxonomic diversity and demonstrate a conserved species membership across most kombucha microbiomes. Though kombucha has previously been characterized as a variable assemblage of yeast, AAB, and LAB (16), our metagenomic survey data, as well these recently published amplicon data, point to a relatively conserved core species membership.

### Synthetic pairwise interaction experiments reveal divergent bacterial responses to yeast.

Metagenomic sequencing approaches can determine the core membership of fermented foods like kombucha, but they cannot reveal the dynamic interactions between community members. Several previous studies have used experimental approaches to characterize pairwise combinations of bacteria and yeast from kombucha, but these previous studies have been unable to consistently recreate the cellulosic biofilms that are a hallmark of typical kombucha fermentations. For example, Liu et al. (14) grew acetic acid bacteria on the autoclaved spent media of kombucha isolates of Saccharomyces cerevisiae, Z. bailii, and B. bruxellensis, but no biofilm formation was reported. Similarly, Tran et al. (12) used defined cocultures of kombucha yeast and AAB and quantified CFU during fermentation of tea, but “no consistent biofilm was produced after 14 days under all conditions.” The inconclusive nature of these previous attempts to deconstruct and reconstruct kombucha biofilms leaves a major gap in our understanding of how different pairwise combinations of kombucha microbes shape the dynamics of this fermentation.

**FIG 2:**
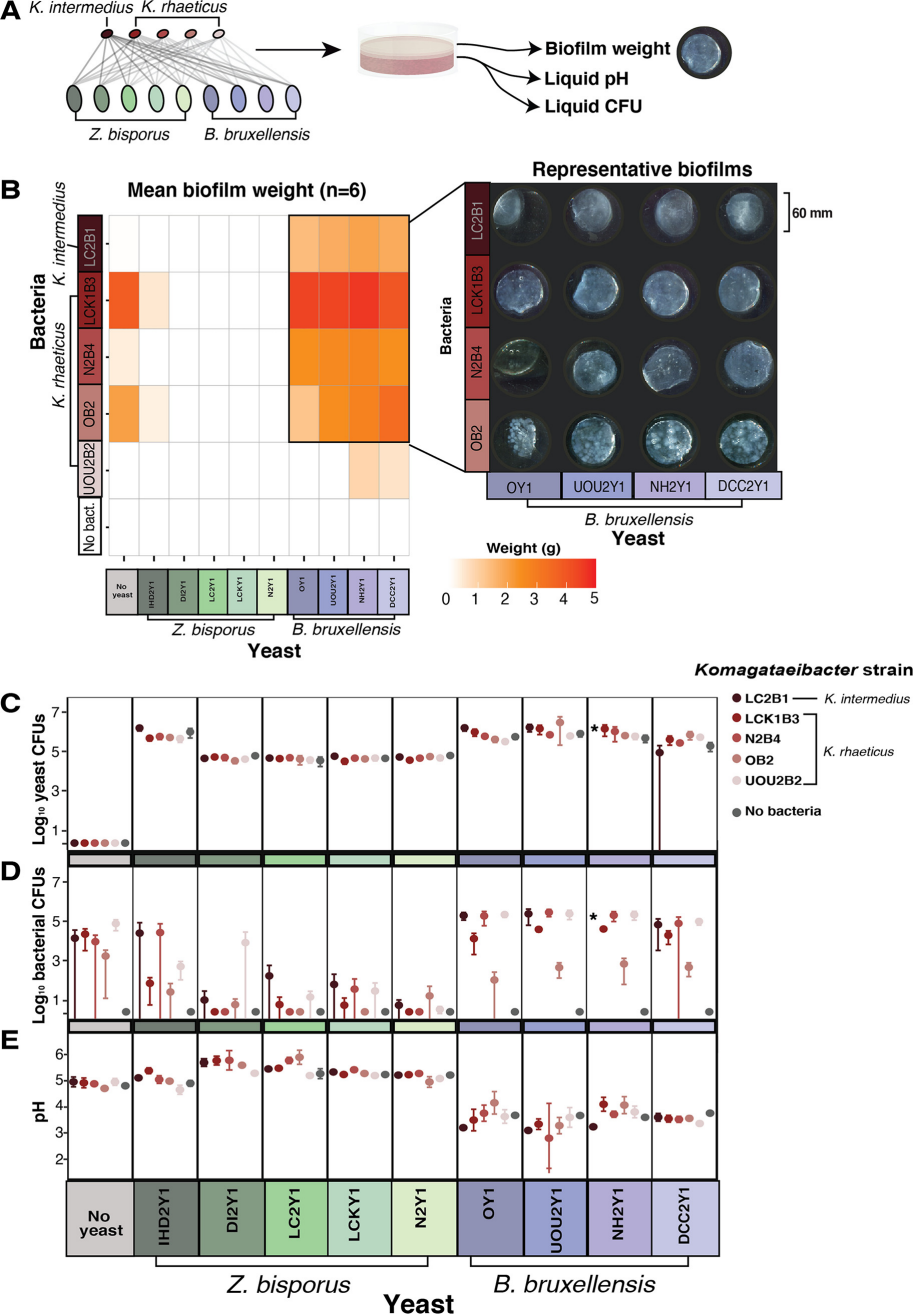
Genus-level differences in yeast, as well as strain-level differences in bacteria, have consequences for kombucha biofilm formation. (A) Experimental design of synthetic pairings of yeast and bacteria. Different color shades of the same yeast or bacterial species represent distinct strains of the species. (B) Biofilm formation measured as wet weight (grams) in synthetic pairs. White indicates no biofilm formation. Select pairs that formed biofilms are linked to images of those biofilms. For images of all biofilms produced, including all replicates, see Fig. S1. (C and D) Log_10_ CFU of yeast and bacteria, respectively, in the liquid portion of synthetic pair fermentations. (E) pH of synthetic pairings at the end of 21 days of incubation. In panels B to E, *n* = 6 replicate fermentations; dots represent the means, and error bars represent the standard deviations of the means. CFU counts from 1 of the 60 experimental treatments—the pair NH2Y1/LC2B1—were not collected due to a plating error during the experimental harvest (indicated by an asterisk [*] in panels C and D).

To comprehensively measure interaction outcomes between the core microbes found in kombucha fermentations, we used strains of the bacterium *K. rhaeticus*, the yeast *B. bruxellensis*, and one other yeast, *Z. bisporus*, isolated from across the kombucha samples described above (see Table S4 for a list of strains used and their kombucha samples of origin). We selected these three species because they represent some of the most commonly reported kombucha taxa based on our work and previous studies. We used two different yeast species for these experiments to determine whether there were divergent fermentation outcomes between *K. rhaeticus* and different yeast species and strains. Although *Z. bisporus* was not frequently identified in our study (it was detected in 15 samples but at <1% abundance), it has been reported as a common inhabitant of kombucha fermentations (8). From our collection, we isolated strains of *B. bruxellensis* (*n* = 4), *Komagataeibacter* spp. (*n* = 5), and *Z. bisporus* (*n* = 5), each from different samples. We created a sterile 10% sucrose green tea medium to approximate the nutrient profile of unfermented sweetened tea, and then we inoculated fully factorial pairwise combinations of yeast and bacteria into the medium and measured biofilm formation (wet weight), pH, and cell counts in the liquid after 21 days (Fig. 2A). We acknowledge that our experimental conditions in petri dishes may not perfectly represent typical kombucha fermentations, and we explain a rationale for our approach in Materials and Methods.

10.1128/msystems.00157-22.6 TABLE S3 Relative abundance of yeasts at the species level across each kombucha ferment. Download Table S3, DOCX file, 0.02 MB. Copyright © 2022 Landis et al. 2022 Landis et al. https://creativecommons.org/licenses/by/4.0/ This content is distributed under the terms of the Creative Commons Attribution 4.0 International license.

10.1128/msystems.00157-22.7 TABLE S4 Strains used in experimental pairings. All strains were isolated from our kombucha collection (see Fig. 1 for metagenomic content of each sample). Species assignments are based on ITS and 16S rRNA Sanger sequencing for yeast and bacteria (see Materials and Methods). Download Table S4, DOCX file, 0.01 MB. Copyright © 2022 Landis et al. 2022 Landis et al. https://creativecommons.org/licenses/by/4.0/ This content is distributed under the terms of the Creative Commons Attribution 4.0 International license.

10.1128/msystems.00157-22.2 FIG S1 Biofilms recovered from all pairwise experiments. Images of scanned biofilms from all replicates and all treatments that produced measurable biofilms are shown. Two replicates were lost during preparation, and they are represented by a black “X” through the image. The brightness and contrast of the images have been adjusted for clearer visualization. Download FIG S1, TIF file, 2.1 MB. Copyright © 2022 Landis et al. 2022 Landis et al. https://creativecommons.org/licenses/by/4.0/ This content is distributed under the terms of the Creative Commons Attribution 4.0 International license.

Under our experimental conditions, the mass of the biofilm was driven by the presence of yeast, the yeast genus, and the bacterial strain utilized in the synthetic pair (Fig. 2B). Weak biofilms were sometimes produced by certain bacterial strains in the control with no yeast and when paired with *Z. bisporus* IHD2Y1. Strikingly, no biofilms were produced when bacteria were paired with the other four strains of *Z. bisporus*. Strains of *B. bruxellensis* produced significantly more biofilm relative to both *Z. bisporus* and the no-yeast control (*χ*^2^ = 166.97, df = 2, *P < *0.001; FDR-corrected *P < *0.001 for all Wilcoxon pairwise comparisons). Bacterial strains also promoted differential degrees of biofilm formation and different biofilm phenotypes (χ^2^ = 82.5, df = 5, *P < *0.001; FDR-corrected *P < *0.05 for all Wilcoxon pairwise comparisons between bacteria, except for N2B4 and LB2B1 and OB2 and N2B4, which were not significantly different [*P > *0.05]). One strain, UOU2B2, lacked strong biofilm formation in all pairs, creating weak and discontiguous phenotypes (see Fig. S1 for images of all biofilms). Strain LCK1B3 formed the highest average mass of biofilms. Clear textural differences were also observed between biofilm-forming bacterial strains, with NB2 forming uniquely “lumpy” biofilm phenotypes (Fig. 2B).

Biofilm formation is made possible by the persistence of bacterial cells, but it is not a measure of bacterial cell abundance. By determining the abundances of viable yeast and bacterial cells when grown alone and in reciprocal pairs, we were able to determine how they affect each other’s growth. It has been hypothesized that yeast-bacterium interactions in kombucha biofilms are mutualistic (17). If yeast and bacteria are stimulated by each other’s presence, they should have elevated CFU relative to the control where they are grown alone.

In our study, yeast CFU in the liquid were not significantly impacted by the presence of bacteria (χ^2^ = 0.16, df = 1, *P = *0.69) or by differences in bacterial strains (χ^2^ = 1.787, df = 5, *P = *0.88) (Fig. 2C). Conversely, bacterial CFU in liquid were significantly different depending on the yeast (χ^2^ = 189.75, df = 2, *P < *0.001) with *B. bruxellensis* stimulating more bacterial growth relative to the control and relative to *Z. bisporus* (*P < *0.01 and *P < *0.001, respectively) (Fig. 2D). Interestingly, bacteria had higher CFU levels in the absence of yeast than when they were paired with *Z. bisporus* (*P < *0.001), suggesting that *Z. bisporus* is either competing with these bacteria for nutrients or inhibiting them.

Together, these results suggest that in our synthetic pairs, bacterial populations in the liquid are strongly affected by yeast presence and the type of yeast. They also demonstrate that yeast populations grow in the absence of bacteria. Rather than a mutualistic relationship where both partners benefit, the *B. bruxellensis*-*Komagataeibacter* pairings studied here appear to be closer to commensal interactions where bacteria benefit, while the yeast is largely unaffected. In this lab representation of kombucha, the *Z. bisporus*-*Komagataeibacter* interactions appear to be ammensal given that the yeast is largely unaffected, but the bacteria are inhibited. It is important to note that the conditions under which these interactions were observed were both highly specific (e.g., only one nutrient concentration was included and no other microbes were present) and different than the nutrient conditions under which kombucha is normally brewed (e.g., petri dishes have a much higher surface area to volume ratio than normal kombucha fermentations). The direction and nature of these symbioses may change under different biological and process parameters and if microbial population densities were measured in the biofilm.

In addition to the production of cellulosic biofilms, acidification is a community-level functional output in kombucha fermentations. The recommended final pH range of finished kombucha ferments is 4.2 to 2.5 (1). In our fermentations, we did not “backslop” (serially transfer) any finished kombucha into our synthetic cultures, so all changes in pH were due to microbial metabolite production within the fermentation time period. The pH only reached typical finished kombucha levels when *B. bruxellensis* was a member of the coculture (average pH of 3.6 in *B. bruxellensis* pairs) (Fig. 2E). Moreover, cultures of *B. bruxellensis* reached this pH range even in the control where no bacteria were present. Though AAB are thought to be the main producers of organic acids in kombucha, including acetic, lactic, gluconic, malic, succinic, and citric acids (12), the presence of *B. bruxellensis* may also be an important and overlooked souring agent in kombucha fermentations. Further work analyzing a broader suite of metabolites in conditions more typical of kombucha fermentations is needed to illuminate the fermentative contributions of *B. bruxellensis*.

### Metabolomic profiling identifies potential mediators of kombucha bacterium-yeast interactions.

**FIG 3:**
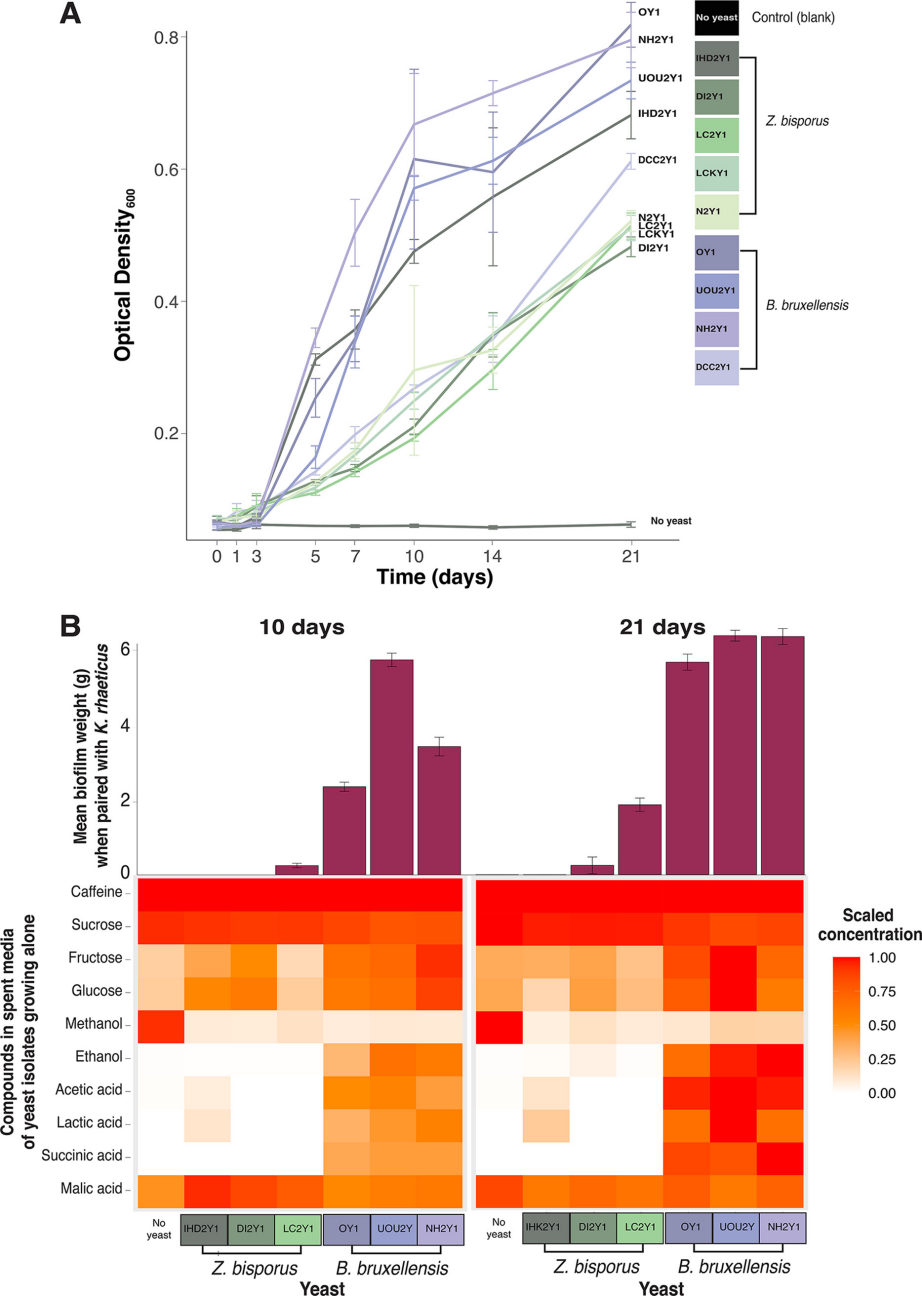
Growth and metabolite profiles of the kombucha yeasts Z. bisporus and *Brettanomyces bruxellensis.* (A) OD_600_ of yeast strains used in synthetic pair experiments growing in 10% (wt/vol) sucrose green tea. Lines show mean values, and error bars represent the standard deviations of the means of seven replicates. (B) NMR analysis of filtered spent media of *Z. bisporus* and *B. bruxellensis* isolates (a subset of those that were used in synthetic pairs) after incubation in 10% sucrose green tea for 10 and 21 days. The concentration of each compound is scaled from zero to the maximum level observed across all measurements (10 and 21 days). A portion of spent medium was also inoculated with K. rhaeticus strain M2B4, and biofilms were weighed. Bacterial biofilm wet weight (g) is displayed as vertical bars, where error bars represent the standard deviations of the means.

The variation in yeast-*Komagataeibacter* interaction outcomes could be explained by yeasts’ differential growth rates, utilization of nutrients in the supplied media, or differential production of metabolites. To further understand yeast growth rates and nutrient profiles that could be driving bacterial growth patterns and fermentation phenotypes, we grew *B. bruxellensis* and *Z. bisporus* strains in our sucrose tea medium for 3 weeks and tracked their growth using optical density (Fig. 3A). We expected that yeast species that did not promote biofilm formation in bacteria would also grow more slowly. At 10 and 21 days, we filtered out cells and analyzed the spent media using nuclear magnetic resonance (NMR) spectroscopy to identify the components that could be stimulating or inhibiting bacterial growth and biofilm formation (Fig. 3B).

Strains of *B. bruxellensis* grew to higher optical densities at the end of 21 days compared to strains of *Z. bisporus* (*F*_2,67_ = 224, *P < *0.001; Fig. 3A) and produced relatively more breakdown products, including fructose, glucose, acetic acid, lactic acid, and succinic acid (Fig. 3B; see Table S5 for ANOVA stats and Tukey values). Sterile tea contained increasing levels of breakdown products fructose and glucose over the two time points. This breakdown is not explained by detectable contamination, since control media were plated on yeast potato dextrose (YPD) and glucose yeast extract agar (GYEA) and no microbial colonies were observed (Fig. 2B). Rather, it may be that sucrose is spontaneously hydrolyzing in tea, which is a phenomenon that has been reported in other studies (12).

10.1128/msystems.00157-22.8 TABLE S5 NMR ANOVA results and *P* values from Tukey *post hoc* tests. Signs of *P* values indicate the direction of differences (positive or negative) between treatments listed in the headers. Caffeine was not detectably different in any treatment. Significant *P* values (<0.05) are marked by asterisks (*, *P* < 0.05; **, *P* < 0.01; ***, *P* < 0.001). Download Table S5, DOCX file, 0.01 MB. Copyright © 2022 Landis et al. 2022 Landis et al. https://creativecommons.org/licenses/by/4.0/ This content is distributed under the terms of the Creative Commons Attribution 4.0 International license.

In addition to the organic acids, one striking difference we observed in the *B. bruxellensis* and *Z. bisporus* metabolite profiles is the ~44 times greater production of ethanol by *B. bruxellensis* compared to *Z. bisporus* (Fig. 3B). Acetic acid bacteria are known to assimilate ethanol as a carbon source, and ethanol is generally thought to stimulate AAB growth and biofilm production in kombucha (12, 13, 16). For example, in a study by Liu et al. (14), the addition of pure ethanol stimulated the growth of AAB type strains. The difference in ethanol production may be one driver of yeast-*Komagataeibacter* interaction outcomes.

To test whether yeast metabolic products alone (in the absence of living yeast cells) could differentially drive bacterial biofilm formation, we also inoculated bacteria into the filter-sterilized spent media from these yeasts and tested for biofilm production (Fig. 3B). For these experiments, yeasts were grown in 50-mL bioreactor tubes and, after 21 days, media were filtered, and metabolite profiles were measured. Filtered yeast media were then added to petri dishes and inoculated with bacteria as with the interaction experiments described above.

In general, bacterial biofilms were larger when bacteria were grown in filtered spent media of yeast rather than when the two were grown concurrently in earlier experiments (χ^2^ = 8.45, df = 2, *P < *0.05). Bacteria grown on 21-day spent media of *B. bruxellensis* grew to significantly higher masses compared to when the bacteria were grown with living cells of yeast (*P < *0.05; Fig. 3B). The spent media of some *Z. bisporus* strains was able to stimulate relatively weak biofilm production, but across the 10- and 21-day spent-medium experiments, *B. bruxellensis* again stimulated more biofilm production relative to the control which did not form biofilms and relative to *Z. bisporus* (χ^2^ = 55.25, df = 2, *P < *0.001). This is consistent with our earlier observations that *B. bruxellensis* stimulates biofilm production of AAB more than *Z. bisporus*, even when the yeasts are grown separately from the bacteria and in different experimental conditions (with a larger volume of 50 mL and a greater surface area to volume ratio). These spent supernatant experiments combined with the NMR data demonstrate that differential metabolite production may drive outcomes of yeast-bacterial interactions in kombucha fermentations.

### Genomic variability among kombucha isolates of *Komagataeibacter rhaeticus*.

Our results from the coculture experiments (Fig. 2) demonstrate that different strains of *Komagataeibacter* spp. have unique phenotypes. To better understand how genomic diversity could help explain the phenotypic diversity we observed, we constructed a pangenome from whole genomes of the four isolates of *K. rhaeticus* used in the experimental interactions, along with three previously published reference isolates. We wanted to determine the genomic diversity of these phenotypically diverse strains and hoped to identify specific genes or gene clusters that might be present or absent in strains with unique phenotypes in our interaction assays.

**FIG 4:**
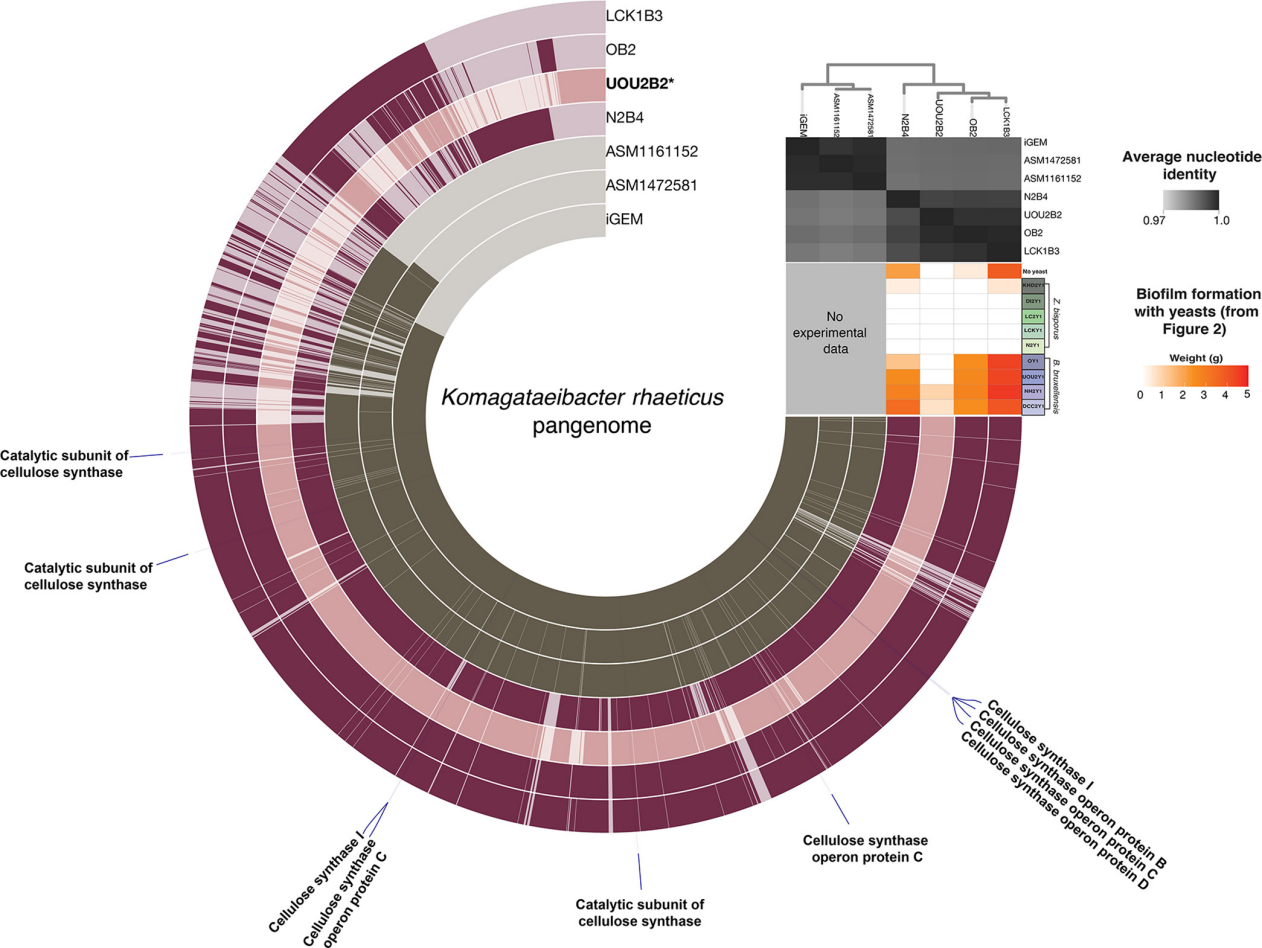
Pangenomic comparison of K. rhaeticus isolates. (A) Pangenome of *K. rhaeticus* isolates from separate kombucha samples, which were used to make defined cocultures of bacteria and yeast. Each layer represents an isolate genome, and each item radiating from the center of the circle is a gene cluster. The presence of a gene cluster in an isolate is indicated by a darker shade of color. On the top right, a dedrogram illustrates similarity of genomes based on hierarchical clustering of the gene cluster presence/absence matrix of all genes from all the genomes. The isolate that forms insubstantial biofilms is highlighted in light pink. Isolates that formed substantial biofilms are in dark pink. Reference genomes are in dark gray. The grayscale heatmap shows the ANI between isolates. The red/orange heatmap originates from Fig. 2 and shows biofilm formation when these bacteria are paired with yeast. Genes annotated as cellulose synthase are labeled in blue.

Overall, bacterial isolates used in our experimental cocultures had >99% average nucleotide identity (ANI) with each other, despite substantial phenotypic differences in biofilm formation (Fig. 4). This pattern has also been found in the closely related cellulose producer *K. xylinus* (15). Our isolate genomes were somewhat distinct from reference genomes in terms of ANI but were still all > 98.5% similar (Fig. 4). This split between our isolates and references could be associated with their discrete geographic origins: all our isolates were collected in the United States, while reference isolates, though also from kombucha, were isolated in Europe. Altogether, the pangenome of these 7 isolates results in 18,174 core genes and 5,041 accessory genes.

Biofilm formation is an important indicator of kombucha fermentation, but cellulose-producing bacteria are also of interest to industrial producers of bacterial cellulose seeking fine-tuned control of this material (15, 18, 19). Kombucha-derived *K. rhaeticus* strains were first identified in 2016 as potential sources for the production of cellulose and are notable for their high cellulose yield at low nutrient concentrations (18, 20). Based on evidence from previously available *Komagataeibacter* genomes, cellulose synthase copy number has been hypothesized as a contributor to cellulose biofilm productivity in *K. rhaeticus* (18). By comparing the isolate that produced minimal biofilms (UOU2B2) to robust biofilm-forming isolates, we did not find evidence for this. All our isolates had either three or four copies of cellulose synthase catalytic subunits that did not correspond with biofilm weight. Frameshift mutations within cellulose synthase operons have been associated with loss of biofilms in *K. hansenii* (21), but in our analysis each gene in the insubstantial biofilm former that was annotated as cellulose synthase (see Materials and Methods) was identical at the amino acid level to the corresponding gene in isolates that formed substantial biofilms, indicating that a mutation in cellulose synthase was not responsible for the difference in biofilm production (see Fig. S2). Finally, though we did identify certain genes that were absent in our low-biofilm-producing strain and present in the higher biofilm strains (see Data Set S1), no clear genetic markers emerged to explain differences in biofilm formation. We acknowledge that our comparison of a limited number of strains may impede our ability to clearly identify genomic regions that correlate with interaction phenotypes. However, this analysis does demonstrate that genomically similar strains of *K. rhaeticus* can have dramatically different kombucha phenotypes.

10.1128/msystems.00157-22.1 DATA SET S1 Distribution of gene clusters across the *K. rhaeticus* strains. Genes clusters that were absent in the low-biofilm-forming strain (UOU2B2) are noted in column C. Download Data Set S1, XLSX file, 3.2 MB. Copyright © 2022 Landis et al. 2022 Landis et al. https://creativecommons.org/licenses/by/4.0/ This content is distributed under the terms of the Creative Commons Attribution 4.0 International license.

10.1128/msystems.00157-22.3 FIG S2 Sequence alignment of the cellulose synthase operon protein B and catalytic subunit of cellulose synthase. The amino acid alignment is from four *K. rhaeticus* strains used in the experiments (LCK1B3, N2B4, OB2, and UOU2B2) and from three reference strains. Download FIG S2, TIF file, 2.2 MB. Copyright © 2022 Landis et al. 2022 Landis et al. https://creativecommons.org/licenses/by/4.0/ This content is distributed under the terms of the Creative Commons Attribution 4.0 International license.

### Conclusions.

Our metagenomic sequencing study determined that *K. rhaeticus* was the most abundant bacterial member of the kombucha fermentations we surveyed and that *B. bruxellensis* was the most common yeast. These results support a very recently published taxonomic survey of kombucha fermentations across the United States (9). *K. rhaeticus* that had >99% ANI resulted in different biofilm phenotypes, both in terms of weight and appearance, indicating that *K. rhaeticus* isolates are not indiscriminately capable of forming robust biofilms and that identification of isolates with high genomic similarity does not guarantee similar phenotypes.

We were able to reconstruct functional kombucha fermentations using just *B. bruxellensis* and *K. rhaeticus.* These cocultures produced biofilms and reached low pH levels, two hallmarks of kombucha fermentation. This suggests that though we detected on average 8.6 microbial species in each kombucha, these frequent inhabitants of kombucha are also capable, without other taxa, of producing a kombucha-like fermentation. In contrast to previous studies that suggest wide variation in kombucha microbial constituency, there appears to be mounting evidence for a core kombucha microbiome, both taxonomically and functionally. This core functional pairing may be of interest to commercial producers of kombucha wishing to create defined kombucha microbial consortia.

Despite our comprehensive and integrated metagenomic, ecological, and metabolomic characterization, there are several important limitations of our work to consider and that should be addressed in future work. First, phenotypic differences in *K. rhaeticus* could be driven by variation in gene expression that would not be revealed by analyzing genomes. Transcriptional regulation of cellulose synthesis in the *Komagataeibacter* genus remains an important knowledge gap that should be further studied through transcriptomic studies of multiple *Komagataeibacter* strains. Second, for our metagenomic sequencing, we only examined one replicate of the liquid portion of fermentation. Additional within-sample replication, especially sampling of the biofilm, may result in different read abundances or added diversity. Third, our experimental conditions represent a single kombucha fermentation environment and do not represent the full spectrum of tea type, sugar concentration, or environmental conditions that can be used to make kombucha. The high surface area to volume ratio of the Petri dishes may have provided an advantage to *B. bruxellensis*, which is known to grow more quickly and produce more metabolites under aerobic conditions (22). Additional work repeating yeast-bacterial interaction assays across the full spectrum of production conditions can reveal other dimensions of ecological diversity in kombucha microbiomes. Finally, we observed differential production of metabolites by the yeasts in our system that may be responsible for promoting cellulose biofilm production by the acetic acid bacteria. However, additional metabolites that were not captured by our NMR analysis may be mediating these interactions. Given the major differences in ethanol production between *B. bruxellensis* and *Z. bisporus* and the well-known importance of this metabolite for AAB growth (23–25), ethanol is a strong candidate, but additional metabolomic analyses and experiments are required to clearly demonstrate the individual and synergistic roles of metabolites in driving kombucha microbial interactions.

The primary focus of our work is to characterize the microbial diversity and dynamics of fermented foods and not necessarily to provide actionable knowledge for home or commercial fermenters. However, these baseline data could be practical. For example, these data suggest that *B. bruxellensis* is highly abundant in home fermentations and may be important for consistent and robust biofilm formation. Future studies of kombucha could reveal both how *in situ* diversity supports function and how geography and fermentation practices shape that diversity.

Kombucha holds promise as a model system to further explore how interspecies interactions can be leveraged to engineer microbiome services. We conducted pairwise interaction assays to ascertain whether interactions between one yeast and one bacterium were sufficient to form biofilms and whether these yeast-bacteria interactions were specific to certain taxa. Moving forward, incorporating more strains, including other abundant yeasts and LAB, could shed light on the multispecies dynamics in these microbiomes, both to understand how pairwise interactions scale to networks of taxa and to engineer these systems for functions such as cellulose production.

## MATERIALS AND METHODS

### Sample acquisition.

Kombucha starters, which were packaged as cellulosic pellicles (SCOBYs), along with liquid kombucha, were purchased from 22 Etsy online sellers from across the United States. SCOBYs were cut and stored with their liquid at −80°C. Isolates of microbes used in experiments and of *K. rhaeticus* used for whole-genome sequencing were cultured nonsystematically from tea by serial dilution on selective media with antibiotics: for AAB, GYEA with cycloheximide (100 mg/L), and for yeasts, YPD with chloramphenicol (50 mg/L). Distinct morphotypes were cultured from each sample, and Sanger sequencing was used for species identification of yeast using primer sets ITS1f and ITS4 (26, 27) and for bacteria using the 16S rRNA primers 27f and 1492r (28, 29). Individual cultures were stored in 15% glycerol at −80°C.

### Synthetic kombucha experiments.

We created a synthetic sucrose tea medium by steeping three bags of Tazo China Green Tips (1.4 oz each) in 850 mL of autoclaved, deionized water heated to ~80°C. After steeping for 10 min, tea bags were removed, and 100 g of DNase- and RNase-free sucrose was added (30). The tea was cooled to room temperature and subsequently filtered through Falcon disposable filter funnels (0.25-μm pore size). Frozen glycerol stocks of isolates were plated onto YPD or GYEA, and colonies were suspended in 1X phosphate-buffered saline (PBS). Because bacterial colonies are cellulosic, they were homogenized before inoculation using a sterile micropestle and by vortexing. Densities were standardized using a spectrophotometer to an optical density at 600 nm (OD_600_) of 0.5. Then, 100 μL of each inoculum (totaling 200 μL where pairs were inoculated) was added to 6.8 mL of tea in 60-mm petri dishes. For controls where isolates were grown alone, 100 μL of sterile PBS was added as the second “pair.” Pairs were incubated in darkness at 24°C for 21 days. At the end of the experiment, synthetic kombuchas were harvested by serially diluting each replicate to 10^−5^ and spot plating onto selective media: GYEA plus cycloheximide and YPD plus chloramphenicol for bacteria and yeast, respectively. Cellulose was removed using sterile wooden dowels and subsequently weighed on an Ohaur Scout Pro laboratory scale. Cellulose pellicles were also scanned on a flatbed scanner at 1,200 lb/in^2^ to record the pellicle appearance (Fig. 2). The pH of each synthetic kombucha was taken with an 89231-608 VWR pH probe using the liquid that remained after the removal of biofilms.

The conditions under which we performed these experiments were optimized to observe differential biofilm formation and may not represent typical kombucha production. Most notably, we performed experiments in petri dishes with a much higher surface area/volume ratio than typical kombucha fermentations. In pilot experiments with larger liquid volumes and taller containers, we observed stochastic sinking of biofilms and the production of additional, layered biofilms at the air-liquid interface. Petri dishes supported consistent biofilm production. We also allowed our experiments to incubate for 21 days. This may be longer than some kombucha fermentations but is within the fermentation time used in other studies (16). In addition, because we would sometimes see the lack of biofilm production in certain treatments, we wanted to use an extended period of time to make sure bacteria had the opportunity to make any biofilms.

### DNA extraction and sequencing.

To concentrate the DNA in kombucha liquid, each sample was centrifuged for 5 min at a 10,000 relative centrifugal force. DNA was extracted from 500 μL taken from the bottom of each tube after centrifugation using a PowerSoil DNA extraction kit (Qiagen, Inc.) according to the manufacturer’s protocol except that, in the final elution step, the sample was eluted in 50 μL rather than 100 μL in order to increase the concentration of DNA.

Genomic (*K. rhaeticus* genomes used for pangenomic analyses below) and metagenomic sequence libraries were prepared using a method previously described (31) using an NEBNext Ultra II FS DNA Library Prep kit (New England Biolabs). Briefly, we followed the manufacturer’s instructions for low-input (<100 ng) libraries. For each product sampled, we fragmented ~100 ng of DNA (in 26 μL) for 20 min using NEBNext Ultra II FS Enzyme Mix. No size selection was used. This fragmented DNA was then used for Illumina adapter ligation and 13 rounds of PCR enrichment with 8-bp index primers (NEBNext Multiplex Oligos for Illumina). Libraries were sequenced at the Tufts University Core Facility on a NextSeq 500 using a paired-end, high-output sequence run with 150 cycles. Only reads from clusters that passed the default quality filter on the Illumina NextSeq 500 were used in downstream analyses.

Metagenomic raw reads were prepared in the following manner: duplicate paired-end reads were first removed using FastUniq (32) and were subsequently adapter and quality-trimmed using Trimmomatic (33) with the following parameters: ILLUMINACLIP:TruSeq3-PE.fa:2:30:10 LEADING:3 TRAILING:3 SLIDINGWINDOW:4:15 MINLEN:36. Kaiju (34) was used to assign taxonomy to reads. To eliminate false positives, a known artifact of read-based taxonomic analysis (35), a conservative 1% abundance threshold was used to define presence/absence of taxa, which biases our analysis to underestimates of richness, especially of rare taxa. On average, Kaiju classified 87% of reads per sample. Kaiju uses a “lowest common ancestor” algorithm to assign the read taxonomy, with more reads assigned to taxa at coarser taxonomic levels. In this study, 97.9% of classified reads were resolved to the level of order, 62% were resolved at the genus level, and 38% were resolved at the species level.

### Spent yeast medium experiments and growth measurements.

For growth measurements and spent media experiments, yeasts were standardized using a spectrophotometer to OD_600_ 0.5 in PBS and inoculated into green tea. For growth measurements, 5 μL of standardized inoculum was added to 195 μL of tea in clear 196-well plates. Control wells were inoculated with 5 μL of PBS. Plates were covered in sterile film, statically incubated at 21°C for 21 days, and destructively sampled at each time point. Wells were mixed using a multichannel pipette to homogenize before each measurement.

For spent media experiments, tea was added to Corning bioreactor tubes (50 mL), with 570 μL of inoculum added to 39 mL of tea. Spent yeast medium was incubated for 10.5 and 21 days. After incubation, yeast cells were removed using Falcon disposable filter funnels (0.45-μm pore size). Bacterial inocula (100 μL of OD_600_ 0.5) were added to 6.8 mL of green tea and 100 μL of PBS and allowed to grow for 3 weeks. Bacteria and yeast were spot plated at the dilutions used for synthetic kombucha experiments as previously described.

### NMR analysis.

Samples were prepared by pipetting 175 mL of kombucha into a 5-mm NMR tube, along with 100 mL of a maleic acid internal standard solution (99.4%; Sigma-Aldrich, St. Louis, MO) at a concentration of 100 mg/mL, equivalent to the addition of 10 mg of internal standard. Next, 450 mL of deuterium oxide (D_2_O; Cambridge Isotope Laboratories, Tewkesbury, MA) was added, and the sample was thoroughly vortexed. ^1^H quantitative NMR (qNMR) data were obtained on a Varian Mercury-300MVX NMR spectrometer equipped with a Varian 5-mm ATB probe and operating at a ^1^H resonance frequency of 299.99 MHz. The spectrum was obtained with a 60° tip angle pulse-width (8 μs), an acquisition time of 4.2688 s, and a relaxation delay of 15 s. Transients (31) were signal averaged with a spectral width of 15 kHz, and we collected 64K digital points. The data were processed using Mestrelab MNova version 14.1.1-24571 (Santiago de Compostela, Spain), and quantitative component analysis was performed utilizing standard qNMR methodologies (36–38).

### Statistics.

All statistical tests were performed in the R environment (39). For synthetic pair experiments, both biofilm weights and log-transformed bacterial and yeast CFU were determined to be nonnormally distributed, so nonparametric Kruskal-Wallis tests were used to determine the effect of bacterial and yeast treatments on these two measurements. Wilcoxon rank sum tests were used for pairwise tests, which were corrected for multiple comparisons by adjusting for the FDR. The effect of spent media on biofilm weights (versus experiments where live cells were paired) was also determined using Kruskal-Wallis tests with experimental treatment (i.e., coinoculation of live cells of bacteria as in Fig. 2 versus 1.5 week spent media versus 3 week spent media as in Fig. 3) as the independent variable. For spent-medium experiments, analyses of variance (ANOVAs) were also used to assess the effects of yeasts on biofilm formation and concentration of compounds detected through NMR, with yeast species (*B. bruxellensis*, *Z. bisporus*, and a no-yeast control) as the independent variables. ANOVAs were also used to compare ODs at 21 days. Following the ANOVAs, Tukey *post hoc* significance testing was used to ascertain the significance of individual pair differences.

### Genomic and pangenomic analysis.

Paired-end Illumina reads of *K. rhaeticus* genomes generated as described above were trimmed using Trimmomatic 0.36 (33) and assembled into draft genomes using SPAdes 3.11.1 with default parameters (40). These draft genomes were then used by the anvi’o (41) snakemake workflows (42, 43) to compute the pangenomes of *K. rhaeticus* with the command “anvi-run-workflow” and the flag “–workflow pangenomics.” Each genome was annotated in anvi’o with NCBI’s COGs (44) and KEGG KOfams (45) using “anvi-run-ncbi-cogs” and “anvi-run-kegg-kofams.” Prokka (46) was also run to annotate the draft genome assemblies, and the annotations were imported into anvi’o. The command “anvi-gen-genomes-storage” was run to store the isolate contigs in an anvi’o database. To compute the pangenome, we ran “anvi-gen-pangenome,” which quantifies gene similarity within and between genomes using NCBI’s BLAST (47) and clusters groups of similar genes using the Markov cluster algorithm (MCL) (48). The pangenome was visualized using “anvi-display-pan.” We used “anvi-summarize” to extract the list of annotations unique to different groups. Genes for cellulose synthesis were identified by their Prokka annotations. Cellulose synthase catalytic subunits were also confirmed by BLAST searches using reference sequences (UniProtKB/Swiss-Prot P37653.3 [49]).

### Data availability.

Raw Illumina sequence data for all metagenomes and genomes have been deposited in the NCBI Sequence Read Archive in BioProject PRJNA833075.
