# Supplementary material for: Microbial Diversity and Interaction Specificity in Kombucha Tea Fermentations
Source: mSystems. 2022 Jun 7;7(3):e00157-22. doi: 10.1128/msystems.00157-22 (PMC9238417; doi:10.1128/msystems.00157-22)
Supplement: TABLE S2 [file msystems.00157-22-st002.docx]

**Table S2** Relative abundance of other bacteria (including lactic acid bacteria [LAB]) at the species level across each kombucha ferment.

|  | Taxon name | | | | | | | | | | | | | | | | | | |
| --- | --- | --- | --- | --- | --- | --- | --- | --- | --- | --- | --- | --- | --- | --- | --- | --- | --- | --- | --- |
| **Kombucha ID** | *Enterococcus faecium* | *Lactobacillus nagelii* | *Lactobacillus ghanensis* | *Lactobacillus plantarum* | *Mycobacterium gastri* | *Candidatus Kryptobacter tengchongensis* | *bacillus coagulans* | *Klebsiella pneumoniae* | *Neisseria meningitidis* | *Streptomyces sp. AgN23* | *Enterococcus faecium* | *Lactobacillus nagelii* | *Lactobacillus ghanensis* | *Lactobacillus plantarum* | *Mycobacterium gastri* | *Candidatus Kryptobacter tengchongensis* | *Bacillus coagulans* | *Klebsiella pneumoniae* | *Neisseria meningitidis* |
| **CQ** | 11.36 | 0 | 0 | 0 | 0 | 0 | 0 | 0 | 0 | 0 | 11.36 | 0 | 0 | 0 | 0 | 0 | 0 | 0 | 0 |
| **CTG** | 1.85 | 0 | 0 | 0 | 0 | 0 | 0 | 0 | 0 | 0 | 1.85 | 0 | 0 | 0 | 0 | 0 | 0 | 0 | 0 |
| **CXT** | 0 | 0 | 0 | 0 | 0 | 0 | 0 | 0 | 0 | 0 | 0 | 0 | 0 | 0 | 0 | 0 | 0 | 0 | 0 |
| **D** | 0 | 0 | 0 | 0 | 0 | 0 | 0 | 0 | 0 | 0 | 0 | 0 | 0 | 0 | 0 | 0 | 0 | 0 | 0 |
| **DI** | 0 | 0 | 0 | 0 | 0 | 0 | 0 | 0 | 0 | 0 | 0 | 0 | 0 | 0 | 0 | 0 | 0 | 0 | 0 |
| **EC** | 14.73 | 0 | 0 | 0 | 26.29 | 0 | 0 | 0 | 0 | 0 | 14.73 | 0 | 0 | 0 | 26.29 | 0 | 0 | 0 | 0 |
| **HQ** | 15.44 | 0 | 0 | 0 | 0 | 3.98 | 0 | 0 | 0 | 0 | 15.44 | 0 | 0 | 0 | 0 | 3.98 | 0 | 0 | 0 |
| **IHC** | 5.44 | 0 | 0 | 0 | 0 | 0 | 0 | 0 | 0 | 0 | 5.44 | 0 | 0 | 0 | 0 | 0 | 0 | 0 | 0 |
| **LC** | 1.83 | 0 | 0 | 0 | 0 | 0 | 0 | 0 | 0 | 0 | 1.83 | 0 | 0 | 0 | 0 | 0 | 0 | 0 | 0 |
| **LCK** | 1.66 | 0 | 0 | 0 | 0 | 0 | 0 | 0 | 0 | 0 | 1.66 | 0 | 0 | 0 | 0 | 0 | 0 | 0 | 0 |
| **LL** | 2.28 | 2.05 | 0 | 0 | 0 | 0 | 0 | 0 | 0 | 2.73 | 2.28 | 2.05 | 0 | 0 | 0 | 0 | 0 | 0 | 0 |
| **MCC** | 0 | 0 | 0 | 0 | 0 | 6.27 | 0 | 0 | 0 | 0 | 0 | 0 | 0 | 0 | 0 | 6.27 | 0 | 0 | 0 |
| **MD** | 0 | 0 | 0 | 1.41 | 0 | 0 | 0 | 0 | 0 | 0 | 0 | 0 | 0 | 1.41 | 0 | 0 | 0 | 0 | 0 |
| **MMM** | 2.38 | 0 | 0 | 0 | 0 | 0 | 8.21 | 0 | 0 | 0 | 2.38 | 0 | 0 | 0 | 0 | 0 | 8.21 | 0 | 0 |
| **N** | 1.57 | 0 | 0 | 0 | 0 | 0 | 0 | 0 | 1 | 0 | 1.57 | 0 | 0 | 0 | 0 | 0 | 0 | 0 | 1 |
| **NG** | 1.38 | 0 | 0 | 0 | 0 | 0 | 0 | 1.58 | 0 | 0 | 1.38 | 0 | 0 | 0 | 0 | 0 | 0 | 1.58 | 0 |
| **NH** | 0 | 0 | 0 | 0 | 0 | 0 | 0 | 0 | 0 | 0 | 0 | 0 | 0 | 0 | 0 | 0 | 0 | 0 | 0 |
| **O** | 0 | 0 | 0 | 0 | 0 | 0 | 0 | 0 | 0 | 0 | 0 | 0 | 0 | 0 | 0 | 0 | 0 | 0 | 0 |
| **QU** | 1.46 | 0 | 0 | 0 | 0 | 0 | 0 | 1.78 | 0 | 0 | 1.46 | 0 | 0 | 0 | 0 | 0 | 0 | 1.78 | 0 |
| **SD** | 4.14 | 0 | 0 | 0 | 0 | 0 | 0 | 0 | 0 | 0 | 4.14 | 0 | 0 | 0 | 0 | 0 | 0 | 0 | 0 |
| **TU** | 2.8 | 4.85 | 0 | 0 | 0 | 0 | 0 | 0 | 0 | 0 | 2.8 | 4.85 | 0 | 0 | 0 | 0 | 0 | 0 | 0 |
| **UOT** | 1.47 | 0 | 0 | 0 | 0 | 0 | 0 | 0 | 0 | 0 | 1.47 | 0 | 0 | 0 | 0 | 0 | 0 | 0 | 0 |
| **UOU** | 3.74 | 0 | 1.78 | 0 | 0 | 0 | 0 | 0 | 0 | 0 | 3.74 | 0 | 1.78 | 0 | 0 | 0 | 0 | 0 | 0 |
| **group** | LAB | LAB | LAB | LAB | bac | bac | bac | bac | bac | bac | LAB | LAB | LAB | LAB | bac | bac | bac | bac | bac |
| **mean** | **3.2** | **0.3** | **0.08** | **0.06** | **1.14** | **0.45** | **0.36** | **0.15** | **0.13** | **0.12** | **3.2** | **0.3** | **0.08** | **0.06** | **1.14** | **0.45** | **0.36** | **0.15** | **0.13** |
