# Supplementary material for: Microbial Diversity and Interaction Specificity in Kombucha Tea Fermentations
Source: mSystems. 2022 Jun 7;7(3):e00157-22. doi: 10.1128/msystems.00157-22 (PMC9238417; doi:10.1128/msystems.00157-22)
Supplement: TABLE S1 [file msystems.00157-22-st001.docx]

**Table S1**Relative abundance of acetic acid bacteria (AAB) at the species level across each kombucha ferment.

|  | Taxon name | | | | | | | | | | | | | | | | | | |
| --- | --- | --- | --- | --- | --- | --- | --- | --- | --- | --- | --- | --- | --- | --- | --- | --- | --- | --- | --- |
| **Kombucha ID** | *Komagataeibacter rhaeticus* | *Komagataeibacter saccharivorans* | *Gluconacetobacter sp. SXCC-1* | *Komagataeibacter intermedius* | *Komagataeibacter xylinus* | *Komagataeibacter hansenii* | *Gluconobacter oxydans* | *Acetobacter pasteurianus* | *Komagataeibacter swingsii* | *Komagataeibacter europaeus* | *Acetobacter senegalensis* | *Acetobacter tropicalis* | *Acetobacter pomorum* | *Komagataeibacter medellinensis* | *Acetobacter musti* | *Komagataeibacter cocois* | *Komagataeibacter diospyri* | *Acidomonas methanolica* | *Komagataeibacter nataicola* |
| **CQ** | 23.11 | 2.7 | 7.79 | 1.08 | 0 | 0 | 0 | 0 | 0 | 0 | 0 | 0 | 0 | 0 | 0 | 0 | 0 | 0 | 0 |
| **CTG** | 1.46 | 1.12 | 0 | 0 | 0 | 0 | 0 | 0 | 0 | 0 | 0 | 0 | 0 | 0 | 0 | 0 | 0 | 0 | 0 |
| **CXT** | 7.27 | 57.64 | 6.74 | 4 | 1.11 | 3.71 | 1.69 | 0 | 0 | 0 | 0 | 0 | 0 | 0 | 0 | 0 | 0 | 0 | 0 |
| **D** | 1.58 | 0 | 0 | 0 | 0 | 0 | 0 | 0 | 0 | 0 | 0 | 0 | 0 | 0 | 0 | 0 | 0 | 0 | 0 |
| **DI** | 5.35 | 83.12 | 2.46 | 0 | 1.03 | 0 | 0 | 0 | 0 | 0 | 0 | 0 | 0 | 0 | 0 | 0 | 0 | 0 | 0 |
| **EC** | 14.15 | 0 | 14.65 | 0 | 1.4 | 3.98 | 0 | 1.18 | 0 | 0 | 0 | 0 | 0 | 0 | 0 | 0 | 0 | 0 | 0 |
| **HQ** | 0 | 0 | 1.3 | 63.73 | 1.87 | 3.63 | 0 | 0 | 0 | 0 | 0 | 0 | 0 | 0 | 0 | 0 | 0 | 0 | 0 |
| **IHC** | 50.23 | 0 | 16.93 | 2.45 | 1.87 | 1.06 | 0 | 1.86 | 0 | 1.5 | 0 | 0 | 1.91 | 1.22 | 0 | 0 | 0 | 0 | 0 |
| **LC** | 5.58 | 0 | 2.16 | 1.33 | 3.54 | 4.4 | 21.87 | 0 | 0 | 0 | 5.54 | 5.35 | 0 | 0 | 0 | 0 | 0 | 0 | 0 |
| **LCK** | 0 | 73.52 | 1.97 | 0 | 1.61 | 3.91 | 0 | 0 | 0 | 0 | 1.45 | 1.51 | 0 | 0 | 0 | 0 | 0 | 0 | 0 |
| **LL** | 1.59 | 11.79 | 1.3 | 0 | 22.21 | 2.83 | 0 | 0 | 2.97 | 1.55 | 0 | 0 | 0 | 0 | 0 | 0 | 0 | 0 | 0 |
| **MCC** | 21.75 | 5.17 | 7.75 | 2.24 | 0 | 2.4 | 0 | 0 | 0 | 0 | 0 | 0 | 0 | 0 | 0 | 0 | 0 | 0 | 0 |
| **MD** | 3.23 | 1.04 | 1.18 | 0 | 0 | 0 | 0 | 0 | 0 | 0 | 0 | 0 | 0 | 0 | 2.43 | 1.96 | 0 | 0 | 0 |
| **MMM** | 16.1 | 31.61 | 6.53 | 4.63 | 1.35 | 4.55 | 0 | 0 | 0 | 0 | 0 | 0 | 0 | 0 | 0 | 0 | 0 | 0 | 0 |
| **N** | 33.23 | 3.46 | 9.94 | 1.62 | 0 | 0 | 0 | 0 | 0 | 0 | 0 | 0 | 1.05 | 0 | 0 | 0 | 0 | 0 | 0 |
| **NG** | 43.93 | 1.34 | 13.21 | 3.71 | 1.35 | 2.97 | 0 | 1.5 | 0 | 1.16 | 0 | 0 | 1.3 | 0 | 0 | 0 | 0 | 0 | 0 |
| **NH** | 15.44 | 0 | 10.02 | 30.93 | 2.93 | 5.4 | 0 | 1.12 | 0 | 0 | 1.25 | 1.22 | 0 | 0 | 0 | 0 | 0 | 0 | 0 |
| **O** | 3.21 | 0 | 1.06 | 0 | 0 | 0 | 0 | 0 | 0 | 0 | 0 | 0 | 0 | 0 | 0 | 0 | 0 | 0 | 0 |
| **QU** | 52.69 | 2.8 | 14.66 | 0 | 1.26 | 0 | 0 | 1.43 | 0 | 1.25 | 0 | 0 | 0 | 1.03 | 0 | 0 | 0 | 1.11 | 0 |
| **SD** | 23.55 | 0 | 8.84 | 3.2 | 33.63 | 0 | 0 | 1.25 | 4.42 | 2.64 | 0 | 0 | 0 | 0 | 0 | 0 | 1.38 | 0 | 0 |
| **TU** | 12.23 | 14.53 | 4.09 | 4.13 | 1.81 | 7.73 | 0 | 0 | 2.47 | 0 | 0 | 0 | 0 | 0 | 0 | 0 | 0 | 0 | 0 |
| **UOT** | 43.77 | 0 | 13.79 | 1.07 | 1.08 | 0 | 0 | 1.14 | 0 | 0 | 0 | 0 | 0 | 0 | 0 | 0 | 0 | 0 | 0 |
| **UOU** | 34.18 | 0 | 10.39 | 8.61 | 8.28 | 6 | 0 | 1.59 | 0 | 1.56 | 0 | 0 | 1.17 | 1.12 | 0 | 0 | 0 | 0 | 1.02 |
| **group** | AAB | AAB | AAB | AAB | AAB | AAB | AAB | AAB | AAB | AAB | AAB | AAB | AAB | AAB | AAB | AAB | AAB | AAB | AAB |
| **mean** | **17.98** | **12.6** | **6.82** | **5.77** | **3.75** | **2.28** | **1.02** | **0.48** | **0.43** | **0.42** | **0.36** | **0.35** | **0.24** | **0.15** | **0.11** | **0.09** | **0.06** | **0.05** | **0.04** |
