# Supplementary material for: Microbial Diversity and Interaction Specificity in Kombucha Tea Fermentations
Source: mSystems. 2022 Jun 7;7(3):e00157-22. doi: 10.1128/msystems.00157-22 (PMC9238417; doi:10.1128/msystems.00157-22)
Supplement: TABLE S3 [file msystems.00157-22-st003.docx]

**Table S3** Relative abundance of yeasts at the species level across each kombucha ferment.

|  | Taxon name | | | | | | | | | | | | | | | | | | |
| --- | --- | --- | --- | --- | --- | --- | --- | --- | --- | --- | --- | --- | --- | --- | --- | --- | --- | --- | --- |
| **Kombucha ID** | *Brettanomyces bruxellensis* | *Pichia membranifaciens* | *Schizosaccharomyces pombe* | *Zygosaccharomyces rouxii* | *Zygosaccharomyces bailii* | *Saccharomycodes ludwigii* | *Saccharomyces cerevisiae* | *Brettanomyces naardenensis* | *Zygosaccharomyces parabailii* | *Zygosaccharomyces mellis* | *Brettanomyces bruxellensis* | *Pichia membranifaciens* | *Schizosaccharomyces pombe* | *Zygosaccharomyces rouxii* | *Zygosaccharomyces bailii* | *Saccharomycodes ludwigii* | *Saccharomyces cerevisiae* | *Brettanomyces naardenensis* | *Zygosaccharomyces parabailii* |
| **CQ** | 27.28 | 0 | 4.63 | 0 | 4.22 | 0 | 2.77 | 0 | 1.58 | 0 | 27.28 | 0 | 4.63 | 0 | 4.22 | 0 | 2.77 | 0 | 1.58 |
| **CTG** | 87.51 | 0 | 0 | 0 | 0 | 0 | 0 | 0 | 0 | 0 | 87.51 | 0 | 0 | 0 | 0 | 0 | 0 | 0 | 0 |
| **CXT** | 6.48 | 0 | 0 | 0 | 0 | 0 | 0 | 0 | 0 | 0 | 6.48 | 0 | 0 | 0 | 0 | 0 | 0 | 0 | 0 |
| **D** | 92.98 | 0 | 0 | 0 | 0 | 0 | 0 | 0 | 0 | 0 | 92.98 | 0 | 0 | 0 | 0 | 0 | 0 | 0 | 0 |
| **DI** | 0 | 0 | 0 | 0 | 0 | 0 | 0 | 0 | 0 | 0 | 0 | 0 | 0 | 0 | 0 | 0 | 0 | 0 | 0 |
| **EC** | 2.71 | 0 | 0 | 3.39 | 0 | 0 | 0 | 0 | 0 | 1.11 | 2.71 | 0 | 0 | 3.39 | 0 | 0 | 0 | 0 | 0 |
| **HQ** | 0 | 0 | 0 | 0 | 0 | 0 | 0 | 0 | 0 | 0 | 0 | 0 | 0 | 0 | 0 | 0 | 0 | 0 | 0 |
| **IHC** | 3.27 | 0 | 0 | 0 | 0 | 1.61 | 0 | 0 | 0 | 0 | 3.27 | 0 | 0 | 0 | 0 | 1.61 | 0 | 0 | 0 |
| **LC** | 28.28 | 0 | 0 | 0 | 0 | 0 | 0 | 0 | 0 | 0 | 28.28 | 0 | 0 | 0 | 0 | 0 | 0 | 0 | 0 |
| **LCK** | 3.63 | 0 | 0 | 0 | 0 | 0 | 0 | 0 | 0 | 0 | 3.63 | 0 | 0 | 0 | 0 | 0 | 0 | 0 | 0 |
| **LL** | 37.4 | 0 | 0 | 0 | 0 | 0 | 0 | 0 | 0 | 0 | 37.4 | 0 | 0 | 0 | 0 | 0 | 0 | 0 | 0 |
| **MCC** | 41.07 | 0 | 0 | 0 | 0 | 0 | 0 | 1.15 | 0 | 0 | 41.07 | 0 | 0 | 0 | 0 | 0 | 0 | 1.15 | 0 |
| **MD** | 76.24 | 1.82 | 0 | 0 | 0 | 0 | 0 | 0 | 0 | 0 | 76.24 | 1.82 | 0 | 0 | 0 | 0 | 0 | 0 | 0 |
| **MMM** | 13.15 | 0 | 0 | 0 | 0 | 0 | 0 | 0 | 0 | 0 | 13.15 | 0 | 0 | 0 | 0 | 0 | 0 | 0 | 0 |
| **N** | 27.96 | 0 | 0 | 2.08 | 0 | 0 | 0 | 1.08 | 0 | 0 | 27.96 | 0 | 0 | 2.08 | 0 | 0 | 0 | 1.08 | 0 |
| **NG** | 15.99 | 0 | 0 | 0 | 0 | 0 | 0 | 0 | 0 | 0 | 15.99 | 0 | 0 | 0 | 0 | 0 | 0 | 0 | 0 |
| **NH** | 17.03 | 0 | 1.19 | 0 | 0 | 0 | 0 | 0 | 0 | 0 | 17.03 | 0 | 1.19 | 0 | 0 | 0 | 0 | 0 | 0 |
| **O** | 89.42 | 0 | 0 | 0 | 0 | 0 | 0 | 0 | 0 | 0 | 89.42 | 0 | 0 | 0 | 0 | 0 | 0 | 0 | 0 |
| **QU** | 8.54 | 0 | 0 | 0 | 0 | 0 | 0 | 0 | 0 | 0 | 8.54 | 0 | 0 | 0 | 0 | 0 | 0 | 0 | 0 |
| **SD** | 2.29 | 0 | 0 | 0 | 0 | 2.02 | 0 | 0 | 0 | 0 | 2.29 | 0 | 0 | 0 | 0 | 2.02 | 0 | 0 | 0 |
| **TU** | 30.3 | 5.14 | 0 | 0 | 0 | 0 | 0 | 0 | 0 | 0 | 30.3 | 5.14 | 0 | 0 | 0 | 0 | 0 | 0 | 0 |
| **UOT** | 25.44 | 0 | 0 | 0 | 0 | 0 | 0 | 0 | 0 | 0 | 25.44 | 0 | 0 | 0 | 0 | 0 | 0 | 0 | 0 |
| **UOU** | 8.02 | 0 | 0 | 0 | 0 | 0 | 0 | 0 | 0 | 0 | 8.02 | 0 | 0 | 0 | 0 | 0 | 0 | 0 | 0 |
| **group** | Yeast | Yeast | Yeast | Yeast | Yeast | Yeast | Yeast | Yeast | Yeast | Yeast | Yeast | Yeast | Yeast | Yeast | Yeast | Yeast | Yeast | Yeast | Yeast |
| **mean** | 28.04 | 0.3 | 0.25 | 0.24 | 0.18 | 0.16 | 0.12 | 0.1 | 0.07 | 0.05 | 28.04 | 0.3 | 0.25 | 0.24 | 0.18 | 0.16 | 0.12 | 0.1 | 0.07 |
