# Supplementary material for: Microbial Diversity and Interaction Specificity in Kombucha Tea Fermentations
Source: mSystems. 2022 Jun 7;7(3):e00157-22. doi: 10.1128/msystems.00157-22 (PMC9238417; doi:10.1128/msystems.00157-22)
Supplement: TABLE S4 [file msystems.00157-22-st004.docx]

**Table S4 – Strains used in experimental pairings.** All strains were isolated from our kombucha collection (see Fig. 1 for metagenomic content of each sample). Species assignments are based on ITS and 16s rRNA Sanger sequencing for yeast and bacteria (see methods).

| **Strain name** | **Kombucha sample origin** | **Species assignment** |
| --- | --- | --- |
| IHD2Y1 | IHD | *Zygosaccharomyces bisporus* |
| DI2Y1 | DI | *Zygosaccharomyces bisporus* |
| LC2Y1 | LC | *Zygosaccharomyces bisporus* |
| LCKY1 | LCK | *Zygosaccharomyces bisporus* |
| N2Y1 | N | *Zygosaccharomyces bisporus* |
| OY1 | O | *Dekkera bruxellensis* |
| UOU2Y1 | UOU | *Dekkera bruxellensis* |
| NH2Y1 | NH | *Dekkera bruxellensis* |
| DCC2Y1 | DCC | *Dekkera bruxellensis* |
| LC2B1 | LC | *Komagataeibacter intermedius* |
| LCK1B3 | LCK | *Komagataeibacter rhaeticus* |
| N2B4 | N | *Komagataeibacter rhaeticus* |
| OB2 | O | *Komagataeibacter rhaeticus* |
| UOU2B2 | UOU | *Komagataeibacter rhaeticus* |
