# Supplementary figures and images for: Microbial Diversity and Interaction Specificity in Kombucha Tea Fermentations
Source: mSystems. 2022 Jun 7;7(3):e00157-22. doi: 10.1128/msystems.00157-22 (PMC9238417; doi:10.1128/msystems.00157-22)

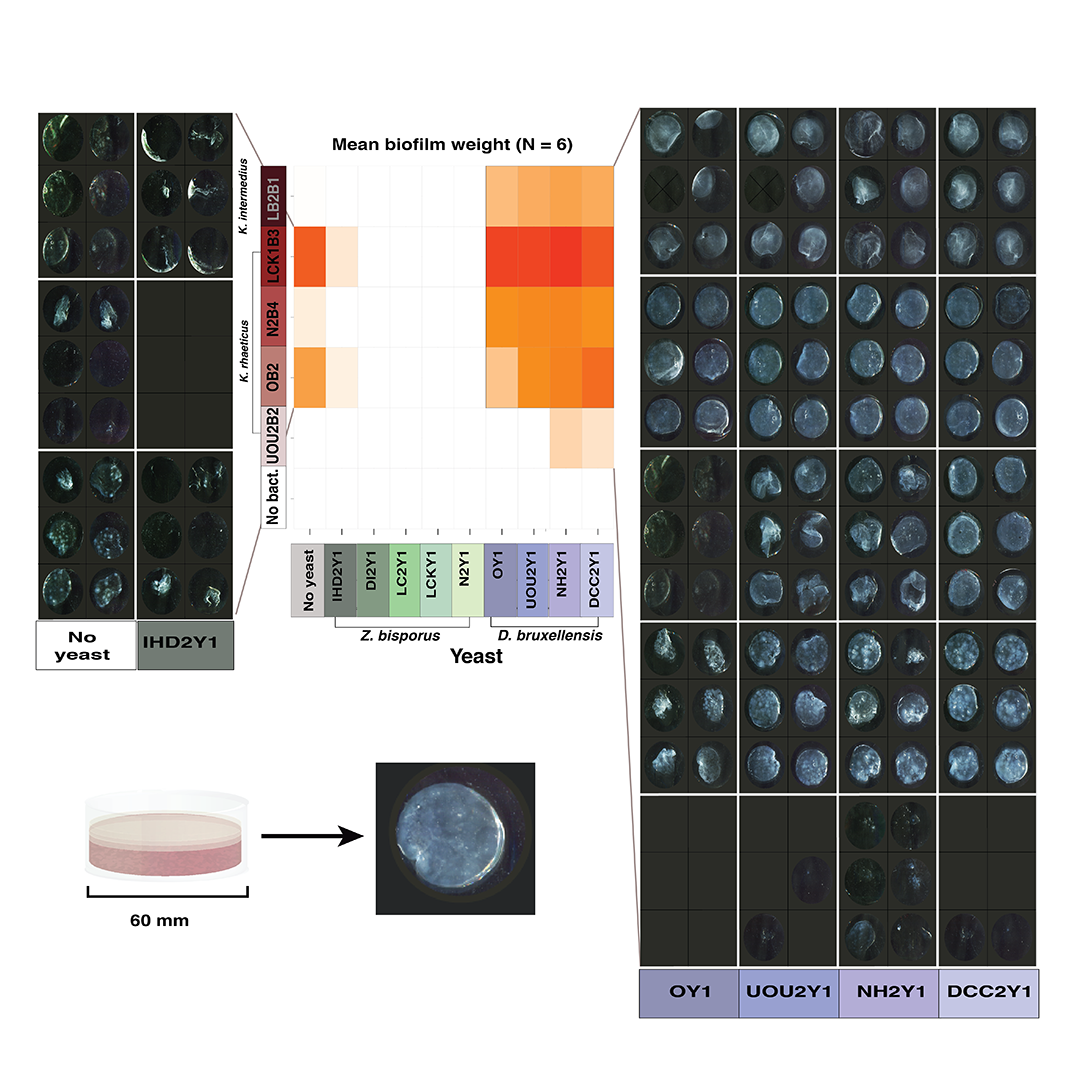

Supplement: FIG S1 [file msystems.00157-22-sf001.tif]

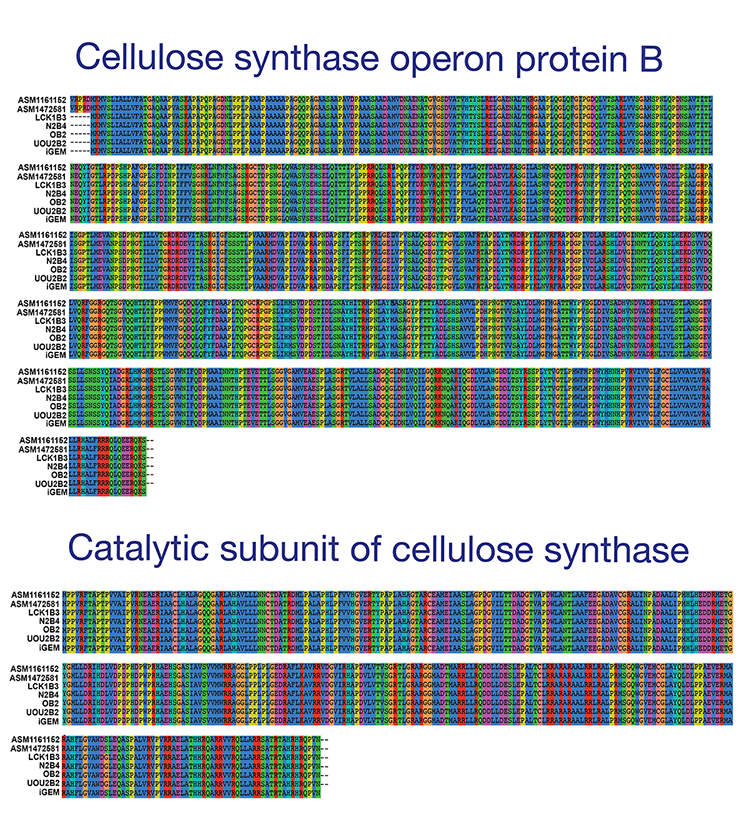

Supplement: FIG S2 [file msystems.00157-22-sf002.tif]
