# Supplementary material for: Microbial Diversity and Interaction Specificity in Kombucha Tea Fermentations
Source: mSystems. 2022 Jun 7;7(3):e00157-22. doi: 10.1128/msystems.00157-22 (PMC9238417; doi:10.1128/msystems.00157-22)
Supplement: TABLE S5 [file msystems.00157-22-st005.docx]

**Table S5 – NMR analysis ANOVA results and *P*-values from Tukey post-hoc tests.** Signs of *P*-values indicate the direction of differences (positive or negative) between treatments listed in the headers. Caffeine was not detectably different in any treatment. Significant *P*-values (<0.05) are marked with asterisks (*<0.05, **<0.01, ***<0.001)

| **Compound** | **ANOVA**  **F-value** | **ANOVA**  **P-value** | **control-*D. bruxellensis:* Tukey P-value** | **control- *Z. bisporus:* Tukey P-value** | ***Z. bisporus-D. bruxellensis*: Tukey P-value** |
| --- | --- | --- | --- | --- | --- |
| **Ethanol** | 38.13 | 0.00248** | - 0.0092554** | - 0.9919786 | - 0.0027660** |
| **Lactic Acid** | 15.95 | 0.0124* | - 0.0340839* | - 0.9203212 | 0.0149115* |
| **Acetic Acid** | 303.2 | 4.29e-05*** | - 0.0001774*** | 0.8742738 | 0.0000638*** |
| **Succinic Acid** | 128.2 | 0.000236*** | - 0.0010016** | 1.0000000 | 0.0002620*** |
| **Fructose** | 16.49 | 0.0117* | - 0.0464088* | - 0.9793405 | 0.0121430* |
| **Glucose** | 7.044 | 0.0489* | - 0.2021269 | 0.8902721 | - 0.0464793* |
| **Sucrose** | 15.26 | 0.0134* | 0.0275130* | 0.6602634 | 0.0191794* |
| **Methanol** | 187.4 | 0.0001** | 0.0001552*** | - 0.2323408 | 0.0001199*** |
| **Caffeine** | --- | --- | --- | --- | --- |
| **Malic Acid** | 3.606 | 0.127 | - 0.2303521 | 0.1131156 | 0.6227935 |
